# Supplementary material for: Neuronal expression in Drosophila of an evolutionarily conserved metallophosphodiesterase reveals pleiotropic roles in longevity and odorant response
Source: PLoS Genet. 2023 Sep 21;19(9):e1010962. doi: 10.1371/journal.pgen.1010962 (PMC10547211; doi:10.1371/journal.pgen.1010962)
Supplement: S1 Table — (DOCX) [file pgen.1010962.s001.docx]

Supplemental Table 1 List of primers used in this study

| **Name of the primer** | **Sequence (5’ to 3’)** |
| --- | --- |
| dMPPED_MfeI_Fwd | GTCAATTGATATCAAAATGGAAGTG |
| dMPPED_XhoI_Rvs | ATCCTCGAGGCATGCTAATCCTTG |
| RP 49_Fwd | CGGATCGATATGCTAAGCTGT |
| RP 49_Rvs | GCGCTTGTTCGATCCGTA |
| CG16717_RT_Fwd | ACATGCCGGCGATTTTACCAAGTGCG |
| CG16717_RT_Rvs | CGAGGATGGACATGCCGGTGTGTTTG |
| Dpt_Fwd | ACCGCAGTACCCACTCAATC |
| Dpt_Rvs | CCCAAGTGCTGTCCATATCC |
| Drs_Fwd | GTACTTGTTCGCCCTCTTCG |
| Drs_Rvs | CTTGCACACACGACGACAG |
| Dro_Fwd | TTTTCCTGCTGCTTGCTTGC |
| Dro_Rvs | TGATGGCAGCTTGAGTCAGG |
| TotM Fwd | TCGACAGCCTGGTCACTTTC |
| TotM Rvs | ACCAAGACCACACGAGCATT |
| Obp28a Fwd | CCGGCCAAGCTAAAGATTGC |
| Obp28a Rvs | CCCCTGAAGCAAGTGCCATA |
| Obp 59 Fwd | TACGGTTACGGAATGGATCACG |
| Obp 59 Rvs | CCGATTGCCTTAGTCCTCGC |
| α-tubulin Fwd | CGAACCAACTAACCTGAGGCTG |
| α-tubulin Rvs | GGCCAATCTGGATGGAGAC |
| furry Fwd | TTGCAGACGCGTATTGTTTTTAGC |
| furry Rvs | TCCAGCTGTTGCGTTGTTGTTG |
| KO specific Fwd | GTGCTCGCATATCTGGCTCTAAG |
| KO specific Rvs | CGGAGAATGAACGACGAGCTT |
| UAS-Reaper Fwd | GACTCTAGCGAGCGCCGGAG |
| UAS-Reaper Rvs | GGCGCAGGGTTTCCAGGACG |
| MPPED2 fwd *NcoI* | GGAGCCATGGCACATGGGATTCCTTCTC |
| MPPED2 rvs *XhoI* | TGCTCGAGTTTRTAGACYKTCCCTCACATTCCAA |
