# Supplementary material for: Neuronal expression in Drosophila of an evolutionarily conserved metallophosphodiesterase reveals pleiotropic roles in longevity and odorant response
Source: PLoS Genet. 2023 Sep 21;19(9):e1010962. doi: 10.1371/journal.pgen.1010962 (PMC10547211; doi:10.1371/journal.pgen.1010962)
Supplement: S2 Table — (DOCX) [file pgen.1010962.s002.docx]

Supplemental Table 2: List of flies used in this study

| Fly line | Source | Identifier | Microinjection site |
| --- | --- | --- | --- |
| *w^1118^* | BDSC | 3605 | NA |
| *IT-gal41111-G4* | BDSC | 65447 | NA |
| *UAS-mCD8-GFP* | BDSC | 5137 | NA |
| *elav-gal4* | BDSC | 458 | NA |
| P{donor} | This study | This study | Random insertion |
| *dMPPEDKO* | This study | This study | NA |
| *UAS-dMPPED* | This study | This study | *attP40* |
| *UAS-MPPED2* | This study | This study | *attP40* |
| *Dredd^EP1412^ (P{w[+mC]=EP}Dredd[EP1412] w[1118])* | BDSC | 10456 | NA |
| *y^1^ w^1118^; P{GawB}GH146* | BDSC | 30026 | NA |
