# Supplementary material for: Neuronal expression in Drosophila of an evolutionarily conserved metallophosphodiesterase reveals pleiotropic roles in longevity and odorant response
Source: PLoS Genet. 2023 Sep 21;19(9):e1010962. doi: 10.1371/journal.pgen.1010962 (PMC10547211; doi:10.1371/journal.pgen.1010962)
Supplement: S1 Fig — (PDF) [file pgen.1010962.s003.pdf]

S1 Fig

Biochemical characterization of dMPPED

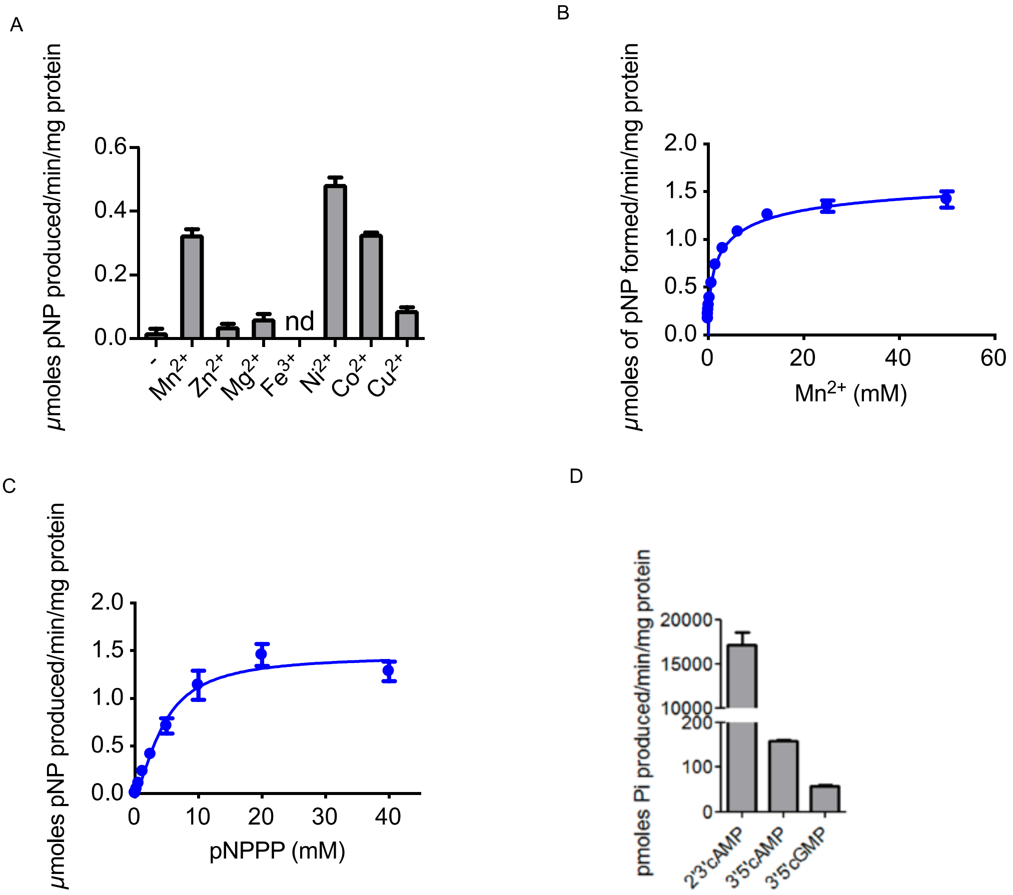

(A**)** Activity of dMpped (500 ng) was assayed in the presence of 10 mM pNPPP and 5 mM of the indicated metals. Values represent the mean ± S.D. of duplicate determinations carried out using two independent protein preparations. (B) dMpped (500 ng) was assayed in the presence of 30 mM pNPPP and varying concentrations of Mn^{2+}. Values represent the mean ± S.D. of duplicate determinations, carried out using two independent protein preparations. (C) dMpped (500 ng) was assayed in the presence of 5 mM Mn^{2+} and indicated concentrations of pNPPP. Values represent the mean ± S.D. of duplicate determinations using two independent protein preparations. (D) Assays were performed with dMppedD (5 μg) in the presence of 1 mM 2'3' or 3'5' cAMP or 5 mM 3', 5' cGMP and 5 mM Mn^{2+}. Free inorganic phosphate released from either 2', 3' or 5' AMP formed following phosphodiesterase-mediated hydrolysis of the cyclic nucleotides was measured by the malachite green assay.
