# Supplementary material for: Neuronal expression in Drosophila of an evolutionarily conserved metallophosphodiesterase reveals pleiotropic roles in longevity and odorant response
Source: PLoS Genet. 2023 Sep 21;19(9):e1010962. doi: 10.1371/journal.pgen.1010962 (PMC10547211; doi:10.1371/journal.pgen.1010962)
Supplement: S2 Fig — (PDF) [file pgen.1010962.s004.pdf]

S2 Fig

Expression of *dMPPED*

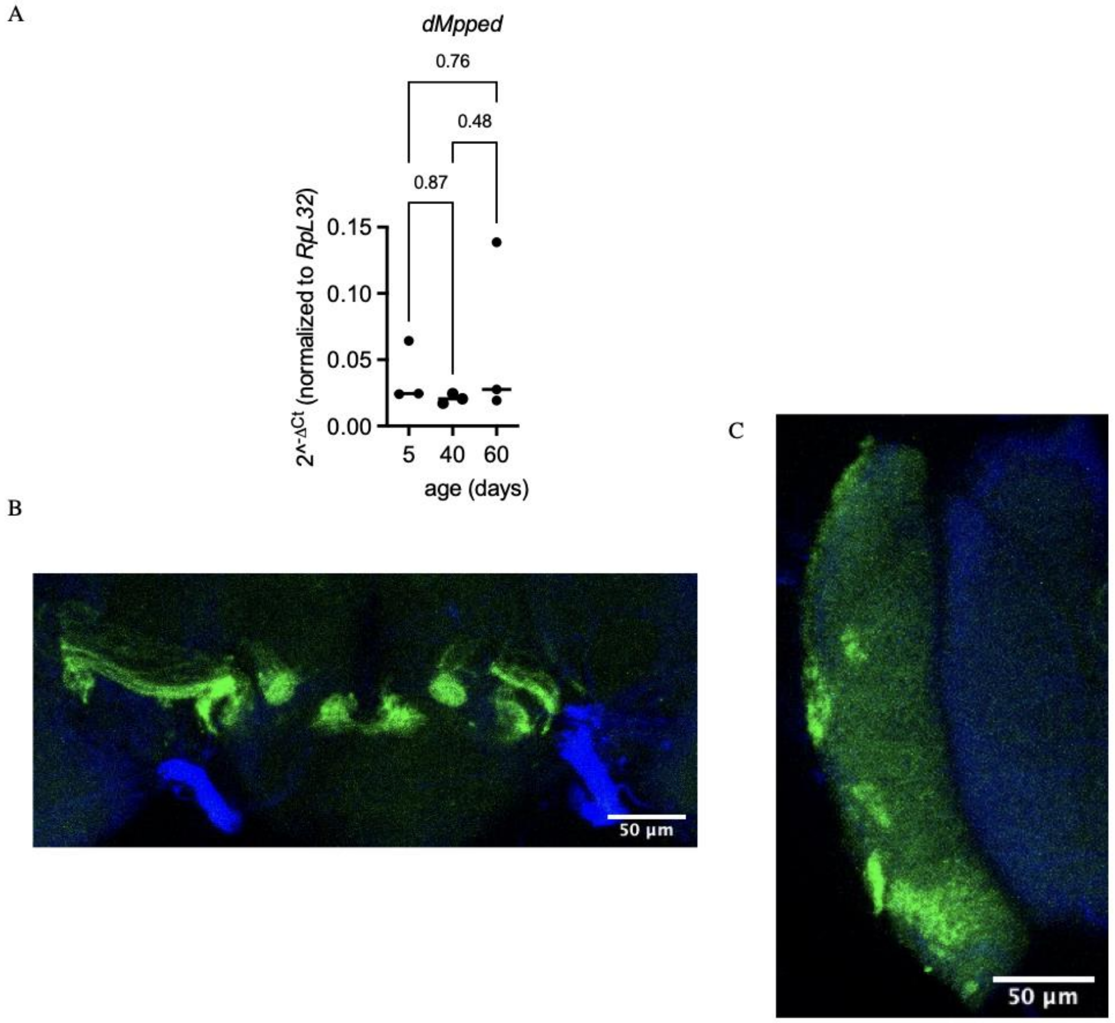

Expression of dMPPED in adult female flies during aging. Values shown are across 3 independent determinations with > 10 flies used in an experiment. B. Enlarged representative images of female brains from *pBAC(IT.GAL4)CG16717/UAS-mCD8GFP* flies. Expression is seen in olfactory projection neurons. C. Enlarged representative images of female optic lobes from *pBAC(IT.GAL4)CG16717/UAS-mCD8GFP* flies.

Supplemental Figure 3
