# Supplementary material for: Neuronal expression in Drosophila of an evolutionarily conserved metallophosphodiesterase reveals pleiotropic roles in longevity and odorant response
Source: PLoS Genet. 2023 Sep 21;19(9):e1010962. doi: 10.1371/journal.pgen.1010962 (PMC10547211; doi:10.1371/journal.pgen.1010962)
Supplement: S3 Fig — (PDF) [file pgen.1010962.s005.pdf]

S3 Fig

Generation and Confirmation of *dMPPED*^{KO} flies

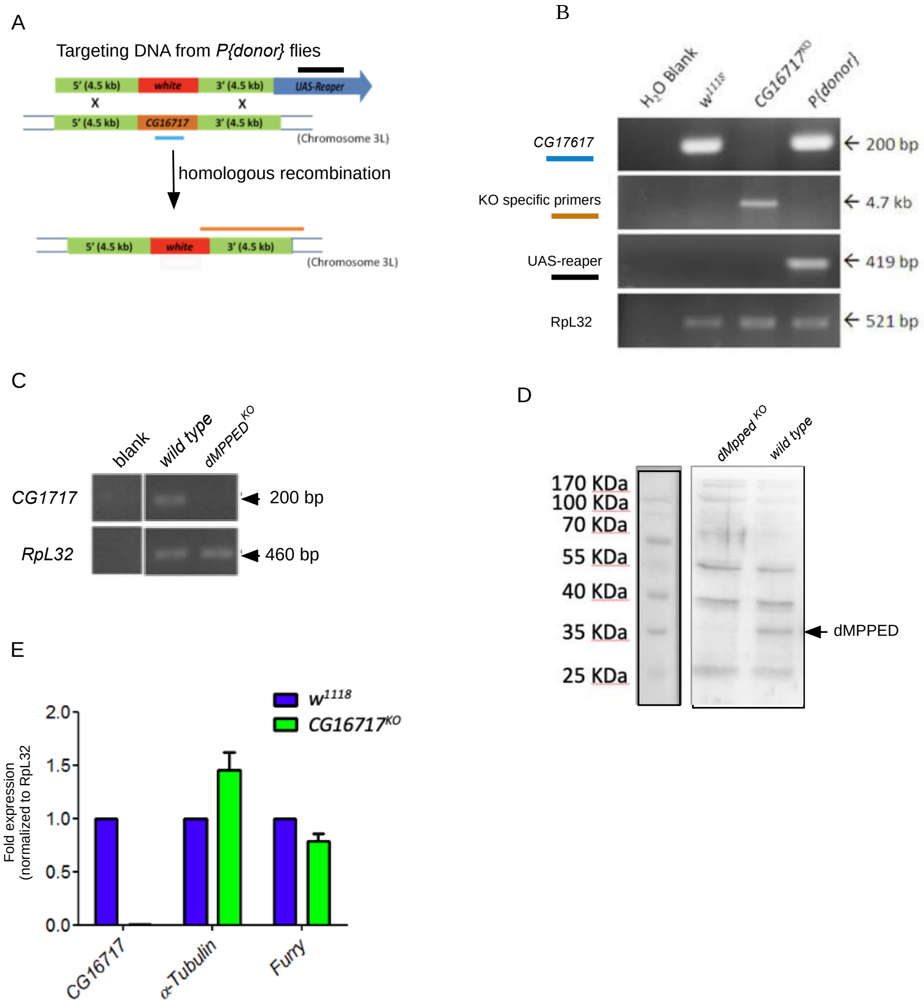

(A) Schematic of homologous recombination at the *dMpped (CG16717)* locus highlighting the positive selection marker white^{+} and negative selection marker UAS-reaper. Various primers used in subsequent PCRs are depicted as blue, orange or black lines. (B) Genomic PCR of *dMpped*^{KO} flies using various primers whose amplicons are color coded with the schematic in (A). *Wild type* (*w*^{1118}) and P[1] flies were used as controls for the PCR. (C) RT-PCR performed on *dMpped*^{KO} flies using *dMpped* specific primers. *RP49* is used as a loading control. (D) Western blot analysis was performed on protein prepared from brains of flies using dMpped-specific antibody. The entire protein obtained from 10 brains of indicated genotypes was loaded in each lane. The blot was stripped and re-probed using the anti-tubulin primary antibody as a loading control. (E) Expression levels of *α-Tubulin 67C* and *Furry* in 4 day-old *wild type* and *dMpped*^{KO} flies. Transcript levels have been normalized to *RP49* and further normalized to the *wild type* flies. Data shown represents mean ± S.D. from 2 independent experiments performed using 10 flies each.
