# Supplementary material for: Neuronal expression in Drosophila of an evolutionarily conserved metallophosphodiesterase reveals pleiotropic roles in longevity and odorant response
Source: PLoS Genet. 2023 Sep 21;19(9):e1010962. doi: 10.1371/journal.pgen.1010962 (PMC10547211; doi:10.1371/journal.pgen.1010962)
Supplement: S4 Fig — (PDF) [file pgen.1010962.s006.pdf]

S4 Fig

Full Blot for Figure 3A

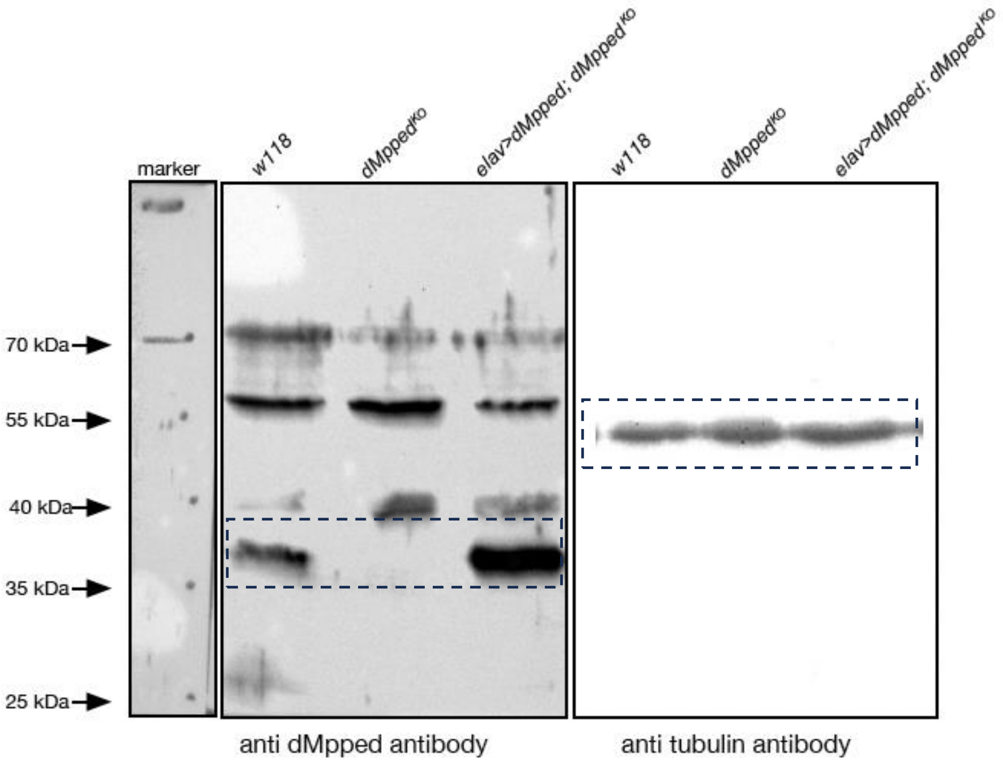

Full blot for Figure 3A showing the absence of a band at 36 kDa representing dMPPED. The blot was normalised to tubulin following stripping and reprobing the blot with an anti-tubulin antibody. The dashed line represents the region shown in Figure 3A.
