# Supplementary material for: Neuronal expression in Drosophila of an evolutionarily conserved metallophosphodiesterase reveals pleiotropic roles in longevity and odorant response
Source: PLoS Genet. 2023 Sep 21;19(9):e1010962. doi: 10.1371/journal.pgen.1010962 (PMC10547211; doi:10.1371/journal.pgen.1010962)
Supplement: S5 Fig — (PDF) [file pgen.1010962.s007.pdf]

S5 Fig

Odorant-binding protein expression in *dMPPED*^{KO} flies.

A

FlyBase id			Gene Name	Log2FC	FDR
FBgn0030985	CG15883	Odorant-binding protein 18a	Obp18a	1.3217285	0.0025632
FBgn0031109	CG11748	Odorant-binding protein 19a	Obp19a	3.7733603	0.0071573
FBgn0011283	CG6641	Odorant-binding protein 28a	Obp28a	5.2609400	0.0000003
FBgn0034468	CG11797	Odorant-binding protein 56a	Obp56a	2.5089205	0.0000001
FBgn0034474	CG13873	Odorant-binding protein 56g	Obp56g	2.2567858	0.0025970
FBgn0034766	CG13517	Odorant-binding protein 59a	Obp59a	4.6648250	0.0000469
FBgn0011281	CG11421	Odorant-binding protein 83a	Obp83a	6.7681838	0.0000043
FBgn0010403	CG11422	Odorant-binding protein 83b	Obp83b	4.7873590	0.0014813
FBgn0039682	CG7584	Odorant-binding protein 99c	Obp99c	0.6571736	0.0027950

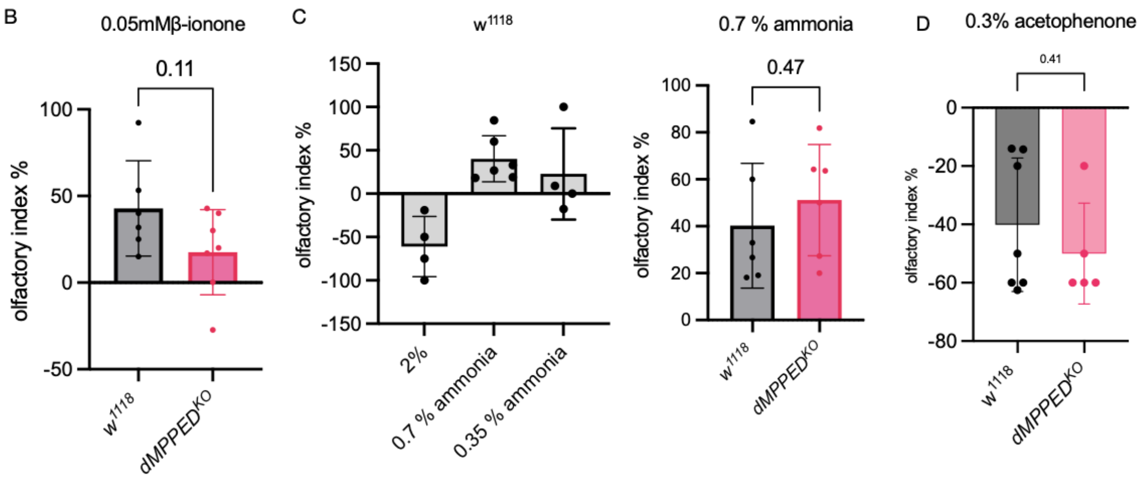

(A) Log2 FC and FDR of Obp specifically upregulated in *dMPPED*^{KO} flies obtained from RNAseq analysis. (B) *w*^{1118} and *dMPPED*^{KO} flies respond similarly to 0.05 mM β-ionone, an attractant. Data is shown from 6 independent trials (*w*^{1118} and *dMPPED*^{KO} flies, 102 of each). Data was analyzed using a t-test. (C) Response of control and *dMPPED*^{KO} flies to ammonia, whose behavioral response is mediated by ionotropic odorant receptors. 0.7% ammonia is an attractant (87 flies across 6 trials), while a concentration of 2% (49 flies across 4 trials) is repellent. Flies show no preference to a concentration of 0.35% (51 flies across 4 experiments). Therefore, 0.7% ammonia was used to test the response of *dMPPED*^{KO} flies. (D) Response of control and *dMPPED*^{KO} flies to a repellent, acetophenone, that is dependent on expression of *Obp56f*, *Obp56h* and *Obp83a* [2], none of which were misregulated in *dMPPED*^{KO} flies. Data shown is from at least 5 trials (*w*^{1118} 69 and *dMPPED*^{KO} 50 flies). Data was analyzed using a t-test.
