# Supplementary material for: Neuronal expression in Drosophila of an evolutionarily conserved metallophosphodiesterase reveals pleiotropic roles in longevity and odorant response
Source: PLoS Genet. 2023 Sep 21;19(9):e1010962. doi: 10.1371/journal.pgen.1010962 (PMC10547211; doi:10.1371/journal.pgen.1010962)
Supplement: S6 Fig — (PDF) [file pgen.1010962.s008.pdf]

S6 FigA

Full blot for Figure 5B

A

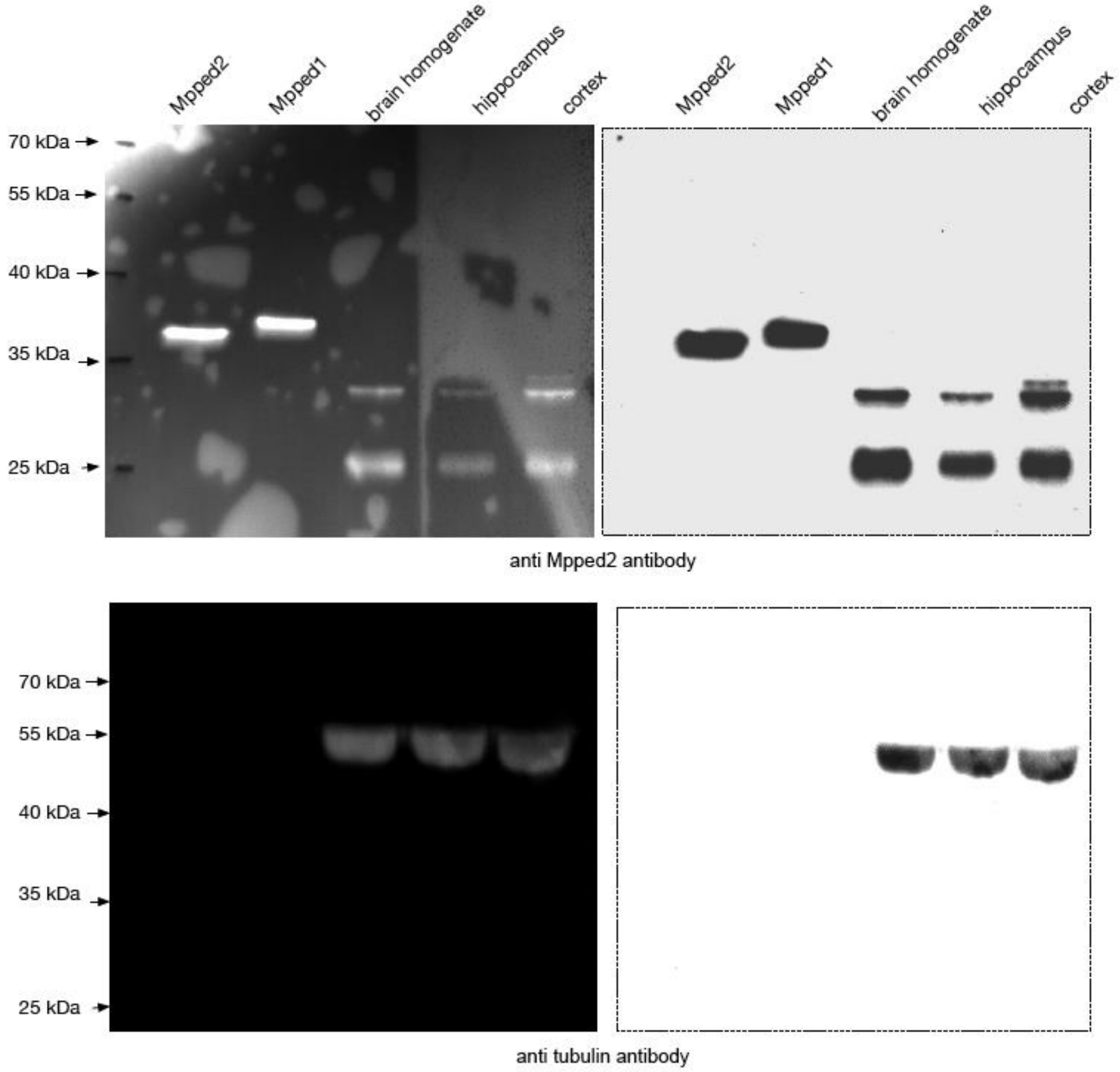


Entire blot for Figure 5B, shown with markers and image captured from the chemiluminescence and the reverse image used in the Figure 5B.

S6 Fig B

B

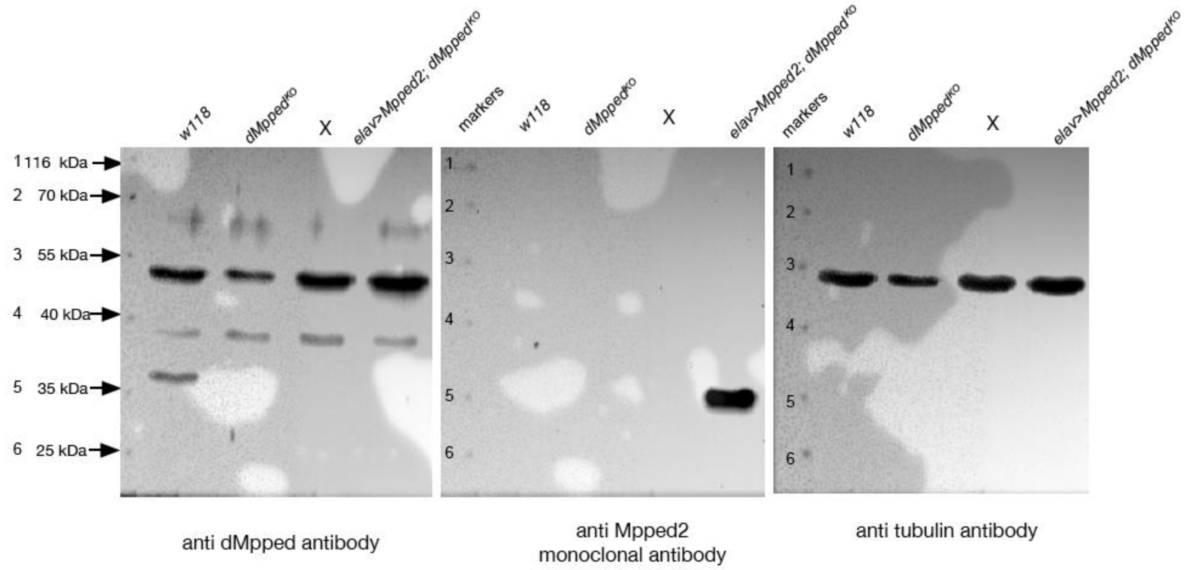


Entire blot for Figure 5E. X represents an irrelevant lane for this Figure. Numbers to the left of the blots refer to the molecular weight markers shown in the leftmost blot.

References


1 Hautakangas, H., Winsvold, B. S., Ruotsalainen, S. E., Bjornsdottir, G., Harder, A. V. E., Kogelman, L. J. A., Thomas, L. F., Noordam, R., Benner, C., Gormley, P., *et al.* 2022 Genome-wide analysis of 102,084 migraine cases identifies 123 risk loci and subtype-specific risk alleles. *Nat Genet*. **54**, 152-160. (10.1038/s41588-021-00990-0)

2 Swarup, S., Williams, T. I., Anholt, R. R. 2011 Functional dissection of Odorant binding protein genes in Drosophila melanogaster. *Genes Brain Behav*. **10**, 648-657. (10.1111/j.1601-183X.2011.00704.x)
