# Supplementary material for: Neuronal expression in Drosophila of an evolutionarily conserved metallophosphodiesterase reveals pleiotropic roles in longevity and odorant response
Source: PLoS Genet. 2023 Sep 21;19(9):e1010962. doi: 10.1371/journal.pgen.1010962 (PMC10547211; doi:10.1371/journal.pgen.1010962)
Supplement: Attachment — Submitted filename: Reviewers Comments 03072023.pdf [file pgen.1010962.s010.pdf]

Reviewer's Responses to Questions

Comments to the Authors:
The review is uploaded as an attachment.

Reviewer #1:

The study is well organized, easy to read and to understand. Overall, it is a high quality piece of work and constitutes a significant advance to the field. Below are some suggestions to the authors to improve the manuscript.

We thank the reviewer for the comments and have incorporated all suggestions to improve the manuscript. Details of the changes are provided below.

Major Comments:The presentation of the statistical analysis is very confusing in the legends. I would very much recommend a detailed description of the statistics in the Material and Methods. For example, we never know if the statistics are performed on the groups or on the individuals. In Figure 4C, the legend is confusing. I assume from the figure that each dot corresponds to an experiment. Hence the legend should only cite the number of experiments, especially since the statistics are supposed to be made on the groups and not the individuals. Also, legends indicate the use of ANOVA for statistics, for the high concentration experiment, you cannot perform ANOVA

We have now included a section in the Materials and Methods called 'Data Analysis' (page 18 line 593) where we describe the statistics used for experiments. A description of the statistics used is provided in the Legends to the Figures as well.

The olfaction acuity experiment at low concentration seems to have twice more data points than at the low concentration (Figure 4C). It would be appropriate to increase the data points of the high concentration experiments to at least 6. Similarly, in Supplemental Figure 4B-C, 3 data points for an olfactory test is very low and the number of experiments ought to be increased.

We have now increased the data points in experiments and with the inclusion of the data from the GH line where expression of dMPPED occurs only in olfactory neurons (see below; new Figure 4), we have moved all the data for odorants that did not show a response to new Supplemental Figure 5.

A section describing the survival assay is missing in the Material and methods. Only survival to desiccation is present.

We have now included details on the survival assay on Page 15 under 'Lifespan' (page 15, line 451).

The authors argue that the expression of dMpedd in possibly very specific neurons alters odorant perception and hygrosensation. Elav-Gal4 is a general driver targeting all neural populations. This argument would be greatly improved if the authors could replicate the odorant perception experiments and the resistance to desiccation in a more specific subset of neurons. For example, by using orco-Gal4 which targets the neural populations of the antennal lobes. Or by using GH-146-Gal4 which targets the OPNs, particularly since these neurons show a high dMpedd expression (Figure 4A).

We thank the reviewer for this excellent suggestion and have included data using the GH-146-Gal4 line in Figure 4C. We show that expression of dMPPED in olfactory projection neurons rescues the aberrant response of dMPPEDKO flies to 0.01mMβ-ionone.

Minor Comments:

Line 79: hydrolyze instead of hydroylze

Line 93: hydrolyze instead of hdyrolyze

Both have been corrected (current lines 78 and 92)

Line 131: Ayer "We noted high expression in the brain". Figure 1E should be cited, not 1D.

This has been corrected. (line 128)

Line 138: Figure 1F should be cited, not 1E

This has been corrected (line 129).

Line 194: The authors forgot to cite Figure 3C

We have cited Figure 3C (line 192).

Line 198: Supplemental Figure 2D does not show that dMpped can hydrolyze cAMP and cGMP. The right citation is "Supplemental Figure 1D"

This has been corrected (line 199).

The olfactory acuity to ß-ionone has been tested at "high" and "low" concentration, and a significant effect was observed only at low concentration (Figure 4C). For the experiment using ammonia and acetophenone, only one concentration has been used. Please describe whether these concentrations should be considered as "low" or "high" and why is there a difference in results depending on the concentration.

Higher concentrations of both ammonia and acetophenone act as repellents and the new data is now shown in a new Supplemental Figure 5.

Figure 1D-E: Add a title saying dMpped expression.

We have indicated the gene being measured.

Figure 1F: Add the genotype.

This has been added to the Figure.

The expression pattern data from Figure 1F is a very interesting data, providing a movie of the full scan would be very useful to the community. It would for example help to identify which antennal lobe glomeruli are stained and thus give a more precise idea of dMpped neural expression.

We are now providing a movie of the full scan as Supplemental movie and have also included a higher magnification images of the male and female brains as requested by Reviewer 2 in Supplemental Figure 1.

Supplemental Figure 2B: The B is missing in the figure.

This has been corrected in new Supplemental 3B.

Figure 2D: I agree that the data shows a "rescue" but you need to show that dMppedKO and Dredd; dMppedKO are significantly different. It would also help the reader to understand that there is a difference by showing statistics in the figure.

This has been done by inclusion of the Dredd life span data and showing the p values across each genotypes.

Figure 3B: Same comment as Figure 2D: I agree that the data shows a "rescue" but you need to show that dMppedKO and elav-Gal4>dMpped; dMppedKO are significantly different. It would also help the reader to understand that there is a difference by showing statistics in the figure.

We have shown statistics in the Figure.

Figure 4A: specify in the title that you are looking at dMpped expression.

The title has been changed.

Figure 4: The titles "A" and "B" are not at the same height, please realign.

This has been corrected.

Figure 4B: The titles "Obp28" and "Obp59" are not at the same height, please realign.

Figure 4D: Same comment as Figure 2D and 3B: I agree that the data shows a "recue" but you need to show that dMppedKO and elav-Gal4>dMpped; dMppedKO are significantly different. It would also help the reader to understand that there is a difference by showing statistics on the figure.

We have shown the new data and also the statistics in the Figure.

Figure 5A: The legend says "Right panel: survival curves of indicated fly lines." This should be removed, there is no survival curve in Figure 5A.

We thank the reviewer for pointing this out and have made the correction.

Figure 5E: Same comment as Figure 2D, 3B and 4D: I agree that the data shows a "recue" but you need to show that dMppedKO and elav-Gal4> Mpped2; dMppedKO are significantly different. It would also help the reader to understand that there is a difference by showing statistics in the figure

We have shown the new data and also the statistics in the Figure.

The authors show that obp expression and so olfaction has an impact on lifespan and suggest a connection with anti-microbial peptide and crystal cell production. It is known that olfaction has a strong impact on hematopoiesis (Shim et al., Cell 2013), could the authors discuss their results in line with this publication?

We have now included a discussion on this finding (reference 63 and page 11, line 337 onwards) and also commented on a report where Obp83b has been shown to play a role in longevity (paragraph on page 12, starting from line 344).

In Figure 4A, the authors analyzed the expression of dMpped in 3- and 30-days old flies. Even though they mention in the discussion that dMpped plays a role in aging (lines 281-282), they never discussed the differences of expression between young and old flies. For example, it seems that there is a strong increase of dMpped expression in peptidergic neurons which are involved in many processes such as metabolism or even regulation of olfactory sensitivity. I think it would provide useful information to discuss such interesting results.

We have included a discussion now on the role of peptidergic neurons in the fly and how expression in older flies could regulate a number of phenomena such as metabolism (page 8, line 220).

Virgin females have been used for experiments (line 394, 455). Do the authors know if the mating status of female flies' influences dMpped levels? Also, are the 35 days old flies used for RNA-sequencing virgin or mated (Figure 4A)? If not, can the authors discuss that the differential expression between 3days virgin female and 35 days old mated females is due to aging and not mating status?

We have used virgin females throughout these studies, including 35-day old flies (page 14, line 430). Studies using mated flies would indeed be of interest and we do not have data on this aspect at this time.

The story is very interesting, and so I think it would help the reader if the authors could provide a schematic of the dMpedd pathway, showing the substrates, reactions, as well as the diverse roles investigated. This could be used as a final fig ure summarizing the story.

We thank the reviewer for this suggestion and have now included a new Schematic as Figure 6.

Reviewer #2:

This work explores the participation of the fly ortholog of the mammalian genes MPPED1/MPPED2, a metallophosphoesterase named dMpped, in longevity, immune response, and physiology. The work is conceptually interesting and brings novel insight into the function of MPPED proteins. The last part characterizing neural expression in mammalian neuros, is a good complement to the work. Overall the work is suitable for PLOS GENETICS, but some revision needs to be performed before is ready for publication.

We thank the reviewer for this appreciation of the study and have modified the manuscripts based on their suggestions.

Major comments
Figure 1, panel F (brain expression), although localization of the label makes sense with what the authors indicate, I recommend using higher magnification images and counterstaining with a neuropil marker to visualize the structures more clearly. Additionally, in the female brain, it seems to be an expression in the optic lobe (in the male brain, there is no labeling in this region, but lamina is absent). Is this protein expressed in photoreceptors or lamina neurons in the visual system? If that is the case, please include this in the description, accompanied by higher magnification images. It is very hard to see localization in the antennal lobe with this magnification and without a proper counterstaining.

We are providing higher magnification of the imaged of the brain and also included Supplemental Movies of the imaging. This coupled with the additional genetic work using the GH line emphasize the role of dMPPED in olfactory neurons. Further work will reveal the role of this protein product in photoreceptors and in the visual system as a whole, and will require additional work outside the scope of this study.

Line 205: Authors propose that neuronally elevated cGMP in dMppedKO flies contributes to the life span regulation in the fly, is there any previous work indicating that cGMP concentration in neurons is linked to lifespan?

We have now cited a few examples of where cGMP may play a role in life-span (page8, lines 220 onwards).

Figure 3: What explains the lifespan differences for control flies between the chart in Figure 2D and 3B? I imagine the sex of the fly, but this should be indicated in the figure legend

All life-spans have been performed with virgin female flies. The difference lies in the fact that flies in Figure 2D were not backcrossed and later data used backcrossed flies which resulted in a slight difference in lifespan. This detail has been indicated in the Legends to the Figures (page 28, line 912).

The specific requirement of neuronal expression of Mpped is intriguing, is this connected with the immune effect, and is this independent of the function in the regulation of the expression of Obps?

We have genetic evidence to show that expression of dMPPED in neurons is required for correct levels of AMP expression. In addition, Obp expression also seems to be correlated with expression of dMPPED in neurons (Figure 4). We expect that there may not be a single pathway that dMPPED is involved in and connections between different pathways remains to be elucidated.

Line 218, the authors indicate that deletion of Obp28 results in a reduced preference for b-ionone. Flies KO for dMpped have increased levels of Obp28, but they also present reduced preference for this molecule. Can the authors elaborate on this observation? Could it be that pleiotropic effects in dMpped mutants somehow mask an expected increase in attraction by b-ionine, given the increase in Obp28 expression in the mutant? It is hard to make conclusions without rescue experiments reducing the expression of Obps in the dMpped KO genetic background, using, for instance a mutation in heterozygosity or a RNAi

We agree that exact mechanisms by which overexpression of Obp28 results in reduced preference for β-ionone is not clear in this study. We at this stage would like to highlight the pleiotropic roles that dMPPED plays in an organism and additional studies in line with what the reviewer has suggested for individual Obps would be needed in future.

Line 236, similarly the authors observe that Obp59a is upregulated in dMpped KO flies. However, the phenotype of the dMpped mutant is the same as the mutant of the Obp59. I would expect something else. Again, a genetic interaction experiment could show that reducing Obp59 directly in the dMpped KO genetic background could modify the phenotype observed.

It is becoming clear in many biological phenomena that the right protein levels are required to bring about a phenotypic effect. Obps may interact with multiple odors, and levels of one Obp may influence the response elicited by another. Since we are testing only a single odor in this experiment, it is not clear whether additional odors from the food etc could influence the overall response of the organism by binding to Obp59. What is apparent though is dMPPED regulates the expression of a number of Obps which may influence the activity of another by interaction with a common receptor or coupled G-protein.

Minor comments
In lines 128 and 133, the authors state that dMpped2 (not dMpped) is expressed in various stages, this refers to a previous name? please explain

We aologise for the error-we should have stated dMPPED and this has been corrected.

Figure 1: Please add a schematic indicating the structure of the dMpped gene with the GAL4 insertion, is the GAL4 in the same direction as the gene?

The schematic has now been added to Figure1G.

Line 300, The authors indicate in their discussion that elevated levels of cAMP and cGMP in dMpped flies could lead to activation of IMD in the fly brain, are there any evidence on the effect of elevated cAMP/cGMP in neurons, and its relation with life span?

We have cited papers where cAMP and cGMP have been suggested to have a role to play in lifespan (page 8, line 202).

Line 312, the authors indicate that a relation between Obp and immunity has been shown outside the nervous system, but how Obp expression in neurons could be related to IMD and life span?

We have now cited a paper (reference 64) where Obp83b has been shown to regulate lifespan. Mechanisms underlying how Obps regulate lifespan are unclear at present.

Line 382, is the temperature of the reaction correct? (or is 37C).

We have corrected the temperature to 37° C (line 418).

Reviewer #3:

Overall, the study presents a useful new genetic model of deficiency for the sole Mpped protein in flies that could bypass the experimental challenges of multiple Mpped homologs in mammals. The data are well controlled and document clear phenotypes that can be mapped to specific cells using Gal4s. The unique roles of dMpped in both longevity and odorant response shed light on the roles of an uncharacterized enzyme, but also have implications in mammalian nervous system dysfunction. However, as the authors' address in the Discussion, there is a notable mechanistic gap between altered cyclic nucleotide metabolism in neurons and changes in the RNA-seq, e.g., AMPs and ORs expression, and which cells these changes actually occur in. Some level of insight into this issue would help strengthen the the study.

We thank the reviewer for the comments. We do agree that exact mechanisms are not apparent at present and further more detailed studies are required to decipher the role of this gene product in apparently diverse pathways.

Major points:
1. The link between cyclic nucleotides and AMP production in dMpped mutants is very unclear and could involve systemic signals between different cell types. AMPs are normally made in the major immune organ, the fat body. Does Mpped loss in neurons lead to cell autonomous production of AMPs by adult brain neurons – which would be unprecedented as far as I am aware – or are the AMP transcripts in the RNA-seq coming from the fragment of fat body located in the head? This info would have a big impact on models of dMpped roles in neurons etc.

It is likely that the AMPs are produced by fat bodies associated with the head since dissection would not have removed them. It is possible the signals from neurons expressing dMPPED to the fat body control production of AMPs by the fat body, and absence of dMPPED in neurons results in misregulation. This has been indicated in the current version of the manuscript (page 11, line 319).

2. In addition, the authors seem to presume that dMpped loss elevates AMPs in the absence of infection (lines 160-161). However, all flies have chronic low-level microbial infections. If they want to claim that dMpped loss is sufficient for AMP expression, they should rear these flies in axenic conditions.

We show AMP production in *dMPPED*^{KO} flies is higher than in control reared identically without any deliberate infection with a pathogen. We have modified the statement to mention only inflammation without commenting on the presence or absence of infection/microbes (page 7, line 164).

3. There is little to no explanation as to why an increase in Obp 28 and Obp59 levels in a dMpped ko mimic those seen in Obp28/Obp59 knockout/deleted flies. What is the explanation for this paradoxical relationship between Obp levels and odorant response? Could the upregulation of these OR mRNAs be an indirect compensatory effect of insufficient cyclic nucleotides to act as second messengers following odorant binding? At a minimum, this paradox needs to be addressed in the text.

We have now addressed these issues in the text and also in comments to Reviewer 2 copied below:

It is becoming clear in many biological phenomena that the right levels of a protein are required to bring about a phenotypic effect. Obps may interact with multiple odors, and levels of one Obp may influence the response elicited by another. Since we are testing only a single odor in this experiment, it is not clear whether additional odors from the food etc could influence the overall response of the organism by binding to Obp59. What is apparent though is dMPPED regulates the expression of a number of Obps which may influence the activity of another by interaction with a common receptor or coupled G-protein.

4. There seem to be distinct differences between the male and female brain staining for dMpped in Figure 1F, but no mention of this within the text. This data suggest possible sex-specific roles for dMpped. This issue is significant, as most experiments use females, or virgin females only, with no mention for the underlying reasons for this decision.

We have now commented on the differential expression seen in male and female flies (page 6, line 141; page 27, line 884, Legend to Figure 1). There are likely to be differences in the behavior of male and female flies, and such studies would, of course, be interesting to do. While we have used virgin females for these studies, we have seen a reduction in the life span in male flies as well (data not shown).

5. The experiments are missing controls for Dredd hypomorphs alone and elav>MPPED2 alone (without dMppedKO in the background). We need to know that rescue is not solely due to the expression of a Dredd hypomorph or MPPED2.

We have included data on Dredd hypomorphs alone in the new Figure 2D.

Elav>MPPED2 would actually represent 'overexpression' of a metallophosphoesterase in w^{118} flies and may not the right control for these experiments. Below is life span data available for female flies to date, since generation of the flies after crossing took some time. We have not included this data in the current version since it would take another month to complete the lifespan and submit the revision. As can be seen below, there is no significant change in the survival of *elav>MPPED2* flies in comparison to w^{118} flies till this point in time, while the $dMPPED^{KO}$ flies have started to die as expected.

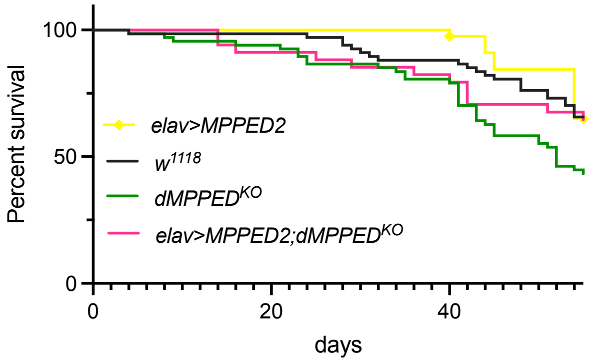

6. Why is the purified protein in Figure 5B running at a different size than the Mpped blotted from the mouse brain? This is not explained in the text and the blot is poorly labelled. Is there a non-specific band present in the lanes from the brain samples?

The different size is because of the hexahistidine tag at the N-terminus of the expressed protein that aids in purification. We have relabeled the blot in the revised version of the manuscript to indicate the two forms of the protein expressed in the mammalian brain (Figure 5; Legend to Figure 5 and page 10, lines 278 onwards).

Other comments
7. The argument that AMP overexpression contributes to reduced lifespan in dMppedKO flies could be strengthened by performing RT-qPCR to assess the levels of the other AMPs mentioned (Drs and Dro) in Figure 2. Considering that RT-qPCR for all AMPs mentioned (Dpt, Dro, and Drs) was performed for conclusions made in Figure 3.

We have shown the levels of Drs and Dro in KO flies in the revised version of the manuscript (new Figure 2E).

8. There is inconsistency in the use of dMpped, CG16717, dMPPED, and dMpped2 within the text and figures and figure legends See line 128,133, Supplemental Figure 2C, Supplemental Figure 2E, Supplemental Figure 4 etc…

We apologize for the inconsistency in the usage of terminology and have checked usage in the revised version.

9. Please highlight H51 and D49 in Figure 1A

This has been done.

10. Separate the inset and the graph into different panels in Fig 1B

This has been done.

11. Missing figure reference in line 113.

We have cited the Figure (line 116).

12. Missing reference in line 132.

We have included the reference in the revised version (line 133).

13. No explanation of why flies were backcrossed for 9 generations for experiments (line 184)

We have included the reason for backcrossing (page 7, line 183 onwards) and in the Materials and Methods section (page 14, line 449) and in Legend to Figure 3. This was done to homogenize the different genetic backgrounds.

14. Define PKG in line 205.

This has been done (page 8, line 206).

15. Format Figure 1 D and E in same manner as other qPCR graphs in the paper

This has been done.

16. Lines 123-124 reference little detectable activity of MPPED2 with Ni2+ and reference data in Supplemental figure

1A, however, these data do not appear in this figure.

We have corrected this statement to clarify that we were referring to MPPED2 which is published data (line 122, reference 9).

17. The blot in Figure 3A for dMpped is not the same blot as in supplemental figure 3, although it is stated that they are the same in the figure legend.

They are the same blot-it is that the one shown in Figure 3A is cropped. We have now clarified this in Supplemental Figure 4 by showing with dashed boxes which region of the whole blot is shown in Figure 3A.

18. Missing reference in sentence on line 201.

We have cited the reference in the revised version.

Have all data underlying the figures and results presented in the manuscript been provided?
Large-scale datasets should be made available via a public repository as described in the PLOS Genetics [data availability policy](), and numerical data that underlies graphs or summary statistics should be provided in spreadsheet form as supporting information.

Reviewer #1: Yes

Reviewer #2: Yes

Reviewer #3: Yes

PLOS authors have the option to publish the peer review history of their article ([what does this mean?]()). If published, this will include your full peer review and any attached files.

If you choose "no", your identity will remain anonymous but your review may still be made public.

Do you want your identity to be public for this peer review? *For information about this choice, including consent withdrawal, please see our [Privacy Policy]().*

Reviewer #1: ***Yes:*** *Dr. Guy Tanentzapf, The University of British Columbia*

Reviewer #2: No

Reviewer #3: No
