# Supplementary material for: Neuronal expression in Drosophila of an evolutionarily conserved metallophosphodiesterase reveals pleiotropic roles in longevity and odorant response
Source: PLoS Genet. 2023 Sep 21;19(9):e1010962. doi: 10.1371/journal.pgen.1010962 (PMC10547211; doi:10.1371/journal.pgen.1010962)
Supplement: Attachment — Submitted filename: Responses to reviewer 2.pdf [file pgen.1010962.s012.pdf]

Responses to reviewers

Reviewer #2: Unfortunately, one my most important comments has not been addressed in this new version. It is clear to me that the authors are presenting data in which the female brain keeps the lamina neuropil (the most lateral neuropil in the optic lobe), which has expression of the driver, while this neuropil is absent in the male brain presented. It is also evident from the Dapi staining that lamina is absent only in males and from the stacks in the supplementary data. Commonly, the lamina is lost during brain dissections for whole mount staining. The reference cited (Rein et al., 1999) indeed describes differences between males and females, but both sexes have the same neuropils, they only differ in size. If the authors want to make the case that male and female have differences in expression of the driver in the optic lobe, they need to present preparations in which brains of both sexes are in the same condition.

We thank the reviewer for these comments and have removed all data on the male brain in the current version (changed Figure 1 and its Legend; changed Supplemental Figure 2 and show only the movie of the female brain). We therefore do not make any comparisons between the male and female brain in this version of the manuscript, which could be an independent course of study in the future.
