# Supplementary material for: Neuronal expression in Drosophila of an evolutionarily conserved metallophosphodiesterase reveals pleiotropic roles in longevity and odorant response
Source: PLoS Genet. 2023 Sep 21;19(9):e1010962. doi: 10.1371/journal.pgen.1010962 (PMC10547211; doi:10.1371/journal.pgen.1010962)
Supplement: Attachment — Submitted filename: Responses to reviewers.docx [file pgen.1010962.s011.docx]

Responses to reviewers

*Reviewer #2: Many of my previous comments have been reasonably addressed in this second version of the manuscript. However, I still feel that the description of the GAL4 line that reports dmpped expression lacks good characterization, the movies provided do not have enough quality, and there is no neuropil counterstaining (e.g. NCadherin, Dlg, Brp, or other) to observe the antennal lobe. The images provided in the supplementary do not allow to precisely define that all the projections are olfactory, and some seem to be gustatory. Furthermore, the female brain, used as an example, keeps part of the lamina in the optic lobe, unlike the male brain. Therefore, it is incorrect to conclude that there is a differential expression in the male vs. female optic lobe because the male does not have part of the brain. Again here, a NCadherin staining would help. The GH 146-GAL4 is expressed in projection neurons in the antennal lobe, among other populations, the authors need to define best if dipped is expressed only in olfactory neurons or also in projection neurons.*

We have now modified the text to be less categorical in defining the specific neurons seen in the Gal4 line. Indeed, further studies would be required to define precisely the neurons in which dMPPED is expressed. We used genetic tools at this time to show that expression of dMPPED in olfactory neurons is important to sense odorants. The counterstaining etc is indeed a good idea and we may follow up on these suggestions in future.

With respect to differences in the male and female brain, we have now cited an additional paper that describes differences between the male and female brain.

*Reviewer #3: Thank you for your efficient efforts in responding to my critiques, and also to those of the other two reviewers. There is an impressive amount of new data in the revision.*

We thank the reviewer for these comments and did indeed add a significant amount of new data in the revised version.

*My final comment/request is that the authors reformat the lifespan data in Figs 2D and 3B. The current format for both undersells the data that is described in the legends. The 2D and 3B have single lines that make it appear that the experiment was done once. Importantly, the figure legends for both panels describe multiple replicates for each genotype, so presumably the single colored lines shown for each genotype is either an average or one representative "run". The data would be more authentic and rigorous if the graphs were shown with error bars for each timepoint, together with a p-value comparing the avg lifespan across all three replicates of each genotype.*

We thank the reviewer for these comments. We did indeed show an average across experiments in the Figure, and the p values do compare the average lifespan across three replicates. We have modified the Figures in 2D and 3B to show error bars and modified the Legends accordingly.
